# Oxidative Stress in Shiga Toxin Production by Enterohemorrhagic *Escherichia coli*

**DOI:** 10.1155/2016/3578368

**Published:** 2015-12-20

**Authors:** Katarzyna Licznerska, Bożena Nejman-Faleńczyk, Sylwia Bloch, Aleksandra Dydecka, Gracja Topka, Tomasz Gąsior, Alicja Węgrzyn, Grzegorz Węgrzyn

**Affiliations:** ^1^Department of Molecular Biology, University of Gdańsk, Wita Stwosza 59, 80-308 Gdańsk, Poland; ^2^Laboratory of Molecular Biology (Affiliated with the University of Gdańsk), Institute of Biochemistry and Biophysics, Polish Academy of Sciences, Wita Stwosza 59, 80-308 Gdańsk, Poland

## Abstract

Virulence of enterohemorrhagic *Escherichia coli* (EHEC) strains depends on production of Shiga toxins. These toxins are encoded in genomes of lambdoid bacteriophages (Shiga toxin-converting phages), present in EHEC cells as prophages. The genes coding for Shiga toxins are silent in lysogenic bacteria, and prophage induction is necessary for their efficient expression and toxin production. Under laboratory conditions, treatment with UV light or antibiotics interfering with DNA replication are commonly used to induce lambdoid prophages. Since such conditions are unlikely to occur in human intestine, various research groups searched for other factors or agents that might induce Shiga toxin-converting prophages. Among other conditions, it was reported that treatment with H_2_O_2_ caused induction of these prophages, though with efficiency significantly lower relative to UV-irradiation or mitomycin C treatment. A molecular mechanism of this phenomenon has been proposed. It appears that the oxidative stress represents natural conditions provoking induction of Shiga toxin-converting prophages as a consequence of H_2_O_2_ excretion by either neutrophils in infected humans or protist predators outside human body. Finally, the recently proposed biological role of Shiga toxin production is described in this paper, and the “bacterial altruism” and “Trojan Horse” hypotheses, which are connected to the oxidative stress, are discussed.

## 1. Introduction: Enterohemorrhagic* Escherichia coli *Strains and Shiga Toxin-Converting Phages

*Escherichia coli* is a bacterial species commonly known as a commensal occurring in the mammalian intestine [1]. This is true in most cases; however, some* E. coli* strains are capable of causing disease in humans. One example of pathogenic* E. coli* is a series of strains called Shiga toxin-producing* E. coli* (STEC) [2, 3].

Among STEC strains (defined as* E. coli* producing Shiga toxins), the most dangerous for humans is the subset classified as enterohemorrhagic* Escherichia coli* (EHEC, defined as* E. coli* causing bloody diarrhea) [2, 3]. Infection of humans by EHEC strains causes hemorrhagic colitis (HC) and in some patients it may result in various complications, including the most severe of them, the hemolytic-uremic syndrome (HUS) [2]. The most common symptoms of this syndrome are acute renal failure, anemia, and thrombocytopenia; however, other organs such as lung, pancreas, and heart may also be affected [4]. Furthermore, some patients suffer from the disorders of the central nervous system [4].

The main virulence factors causing EHEC-mediated HUS are Shiga toxins, produced by the infecting bacteria. These toxins are hexameric proteins, composed of a single A-subunit and five identical B subunits [5]. The main receptor, called Gb3 and occurring on the surface of many types of eukaryotic cells, is recognized by the B-subunits. The toxin enters cells by endocytosis, which is followed by its retrograde transport from the early endosome through the Golgi-apparatus and to the endoplasmic reticulum. The specific proteolytic cleavage of the A-subunit results in the release of the A1 polypeptide from the A2 fragment attached to the B pentamer. A1 is the actual toxin that is translocated from the ER to the cytoplasm [6, 7]. The Shiga toxin A1 polypeptide is an N-glycosidase that depurinates a single adenine residue (A4324) within the *α*-sarcin/ricin loop of the 28S rRNA [8, 9]. This modification results in an inhibition of amino-acyl-tRNA binding to the ribosome and cessation of protein synthesis, which leads to cell death [5].

Since cattle are resistant to Shiga toxins, due to the lack of the Gb3 receptor, they serve as a natural reservoir of STEC strains. However, any cattle-derived products contaminated by STEC, and particularly EHEC, may cause severe human infections, occurring usually as outbreaks. A few years ago (in 2011) such an outbreak took place in Germany, where over 4,000 patients developed severe symptoms and 54 of them died [10–15]. Contaminated fenugreek and lentil sprouts were recognized as the source of the infection [13], indicating that unwashed or improperly washed vegetables, especially those coming from the so-called “ecological farming” where only natural fertilizers (including those coming from cattle) are used, may be a significant source of such outbreaks. In that case, the strain of the O104:H4 serotype, which caused the outbreak, had a combination of virulence factors characteristic of enteroaggregative* E. coli* (EAEC) and EHEC [12, 14]. The high virulence of this particular strain could be ascribed to enhanced adhesion, survival adjustment, antibiotic resistance, and Shiga toxin production [12].

**Figure 1:**
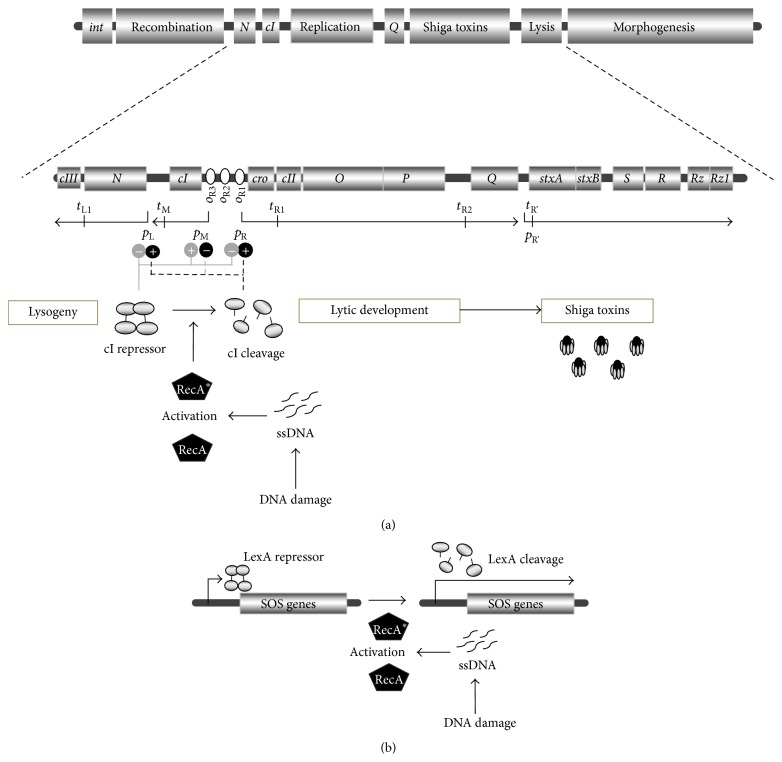
Schematic map of a Shiga toxin-converting phage genome. At the top of (a), regions bearing genes for particular phage functions are shown (as they appear in a prophage). The region containing genes involved in regulation of phage development, DNA replication, Shiga toxin production, and cell lysis is enlarged and shown in more detail. Major transcripts are shown by arrows, with arrowheads demonstrating directionality of transcription, and promoters marked by short vertical lines at the beginning of transcripts. Terminators are marked by vertical lines crossing the transcript lines. The cI repressor binds to *o*_R1_, *o*_R2_, and *o*_R3_ operator sites, repressing *p*_L_ and *p*_R_ promoters and stimulating its own promoter *p*_M_. When DNA is damaged, single stranded DNA (ssDNA) fragments appear which are recognized by RecA protein. This activates RecA to switch to the RecA^*∗*^ form, able to stimulate self-cleavage by the cI repressor. Inactivated cI can no longer repress *p*_L_ and *p*_R_ and *p*_M_ is not activated. This leads to effective transcription from *p*_L_ and *p*_R_, prophage excision, and expression of vast majority of phage genes, including those coding for Shiga toxin. (b) represents a similar mechanism leading expression of the SOS regulon which under normal growth conditions is repressed by the LexA protein. Phage cI repressor resembles LexA; thus under conditions of the SOS response, induction of the prophage occurs.

Interestingly, genes coding for Shiga toxins (*stx* genes) are located in genomes of prophages rather than in actual bacterial genome [16, 17]. Bacteriophages bearing the* stx* genes, called Shiga toxin-converting phages or Stx phages, can lysogenize* E. coli* strains making them STEC. All Stx phages described to date belong to the family of lambdoid phages, viruses having genomes organized in a manner similar to that found in bacteriophage *λ* [17]. The genome of a lambdoid phage consists of blocks of genes coding for proteins responsible for specific functions. This makes recombination and exchange of genes between various phages relatively easy and leads to mosaicism of genomes of lambdoid phages [18]. In genomes of Shiga toxin-converting phages, the* stx* genes are present between the *Q* antiterminator gene and the genes coding for proteins causing cell lysis (Figure 1(a)).

**Figure 2:**
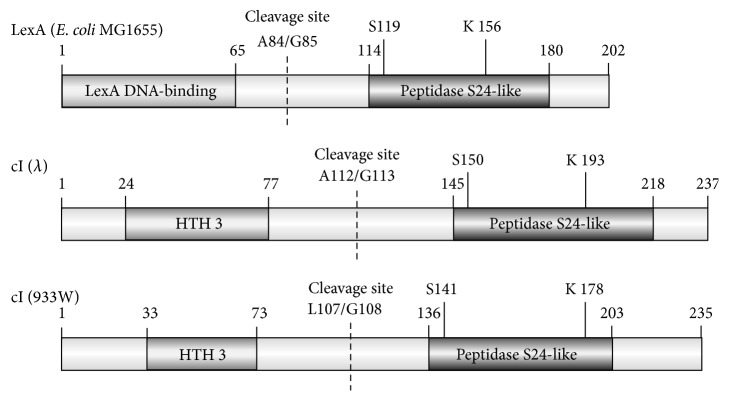
Comparison of domain structures of* E. coli* LexA protein and cI repressors of bacteriophage *λ* [19] and Shiga toxin-converting bacteriophage 933W [23, 24]. Two domains of these proteins are shown, and crucial amino acid residues are marked. Upon stimulation by the activated form of RecA (RecA^*∗*^) both LexA and cI cleave their own molecules (at indicated positions) by the peptidase S24-like domains. The models were prepared using the DOG 1.0: Illustrator of Protein Domain Structures software [25].

As long as the Stx bacteriophage is present in the* E. coli* host as a prophage, vast majority of its genes, including* stx* genes, are silent due to the repression caused by the phage-encoded cI protein [19–21]. Under such conditions, Shiga toxin is not produced. Effective expression of* stx* genes, together with all genes required for lytic development of the bacteriophage, occurs only after prophage induction, though Shiga toxin 1 may also be produced under conditions of low iron levels due to the presence of the Fe-sensitive promoter upstream of the* stx1* locus [17, 20]. The prophage induction occurs generally due to activation of the bacterial SOS response which is a defensive mechanism provoked by any conditions causing appearance of single-stranded DNA fragments. The RecA protein recognizes such fragments and is activated to stimulate the self-cleavage of the LexA repressor (bearing the peptidase domain in its structure), which under normal conditions inhibits expression of the SOS regulon (Figure 1(b)). However, the phage cI repressor resembles LexA (Figure 2) and it is also degraded under the SOS stress response, causing derepression of bacteriophage promoters, excision of the prophage, and subsequent lytic development of the virus. Importantly, in the case of Stx phages, expression of the* stx* genes proceeds together with other phage genes [17, 20, 21] (Figure 1(a)). It is worth mentioning that RecA-independent induction of Shiga toxin-converting prophages by chelating agents, like EDTA, has also been reported [22]. In conclusion, production of Shiga toxins requires induction of Stx prophages, caused by either any stress conditions provoking the SOS response or by chelating agents.

Under laboratory conditions, induction of lambdoid prophages is relatively easy, and standard methods for the efficient induction include UV-irradiation and treatment with antibiotics that interfere with bacterial DNA replication, like mitomycin C [20, 21]. Such treatments lead to prophage excision in a large fraction, if not most, of lysogenic cells in a bacterial population. Nevertheless, when infection of humans by EHEC is analyzed, one should consider prophage induction conditions which can naturally occur in human intestine. Obviously UV-irradiation is very unlikely there, and high concentrations of antibiotics may be administered only to patients subjected to intensive therapy, while symptoms of EHEC infection appear also in nontreated persons. Moreover, other inducers of prophage excision, like EDTA [22], irradiation with ^60^Co [27], or high hydrostatic pressure [28], are also unlikely to occur in the human gut. Therefore, an important question arose: what are factors or agents that can induce Shiga toxin-converting prophages in EHEC-infected human intestine? Understanding the mechanism of stimulation of Shiga toxin production might lead to development of novel methods for prevention or treatment of EHEC-caused diseases, as well as deciphering a biological role for maintaining the Stx prophages in bacterial genomes.

## 2. Hydrogen Peroxide as an Inducer of Shiga Toxin-Converting Prophages

There were various attempts to find conditions which both induce Stx prophages and are likely to occur in the human gut. Different conditions, factors, and agents (including high and low temperatures, high salt concentrations, chelators, ^60^Co, high hydrostatic pressure, nitric oxide, and starvation) were tested, and the results of these studies have been summarized [17]. Most of the tested conditions either did not induce lambdoid prophages or were unlikely to occur in human intestine.

Under conditions of bacterial infection, including infection of the human gut, neutrophils are the first cells of the immune system which attack the pathogens. Among other bactericidal mediators, neutrophils excrete hydrogen peroxide to weaken bacterial cells. This oxidative stress-inducing agent is dangerous for bacteria that are much more sensitive to it than eukaryotic cells. However, it was demonstrated that such an action of neutrophils enhances production of Shiga toxins by EHEC strains [29]. Subsequent studies indicated that hydrogen peroxide, when added to cultures of bacteria lysogenic for various Shiga toxin-converting phages, is a potent inducer of the prophages [30]. This was true for bacteriophage *λ* as well as for different Stx phages. Moreover, the prophage induction was accompanied by synthesis of considerable amounts of the fusion protein, encoded by a gene located in the place of the natural *stx* locus [30]. Very similar results were obtained when natural isolate of EHEC was tested instead of laboratory strains. Again, hydrogen peroxide-mediated induction of the Stx prophage and efficient production of Shiga toxin were observed [31]. Therefore, the oxidative stress, mediated by hydrogen peroxide, leads to stimulation of expression of *stx* genes. Since such conditions can occur in human intestine, the oxidative stress is a likely candidate for a natural inducer of Shiga toxin-converting prophages.

An interesting observation in studies on both laboratory Stx lysogens and natural isolates of EHEC was that the maximal efficiency of prophage induction occurred at a final H_2_O_2_ concentration of 3 mM, and further increases in H_2_O_2_ concentrations caused a decrease in induction efficiency [30, 31]. Moreover, while prophage induction by UV-irradiation or mitomycin C caused a lysis of bacterial cultures in a few hours, no such phenomenon could be detected after treatment with hydrogen peroxide [30]. Subsequent calculations of the efficiency of prophage induction have shown that while low concentration (1 *μ*g/mL) of mitomycin C caused initiation of the lytic phage development in about 10–30% of cells (depending on the kind of the Stx phage), the value of this parameter was as low as 0.03–1.6% at the optimal (for prophage induction, i.e., 3 mM) concentration of hydrogen peroxide [32]. Therefore, in H_2_O_2_-treated lysogenic bacteria, only a very small fraction of cells (usually less than 1%) is induced for prophage excision and subsequent lytic development. Since the rest of bacterial population in the culture can grow and propagate due to resistance to infection by the same phage as the prophage present inside the cell, it is not possible to observe culture lysis.

Another question was what is the mechanism causing the low efficiency of prophage induction under conditions of oxidative stress? Studies on bacteriophage *λ*, the best known representative of lambdoid phages, indicated the factor responsible for such a phenomenon. Since DNA sequences of the regulatory regions of *λ* and Stx phages are very similar [33], one can suppose that the processes occurring in these phages are generally the same.

**Figure 3:**
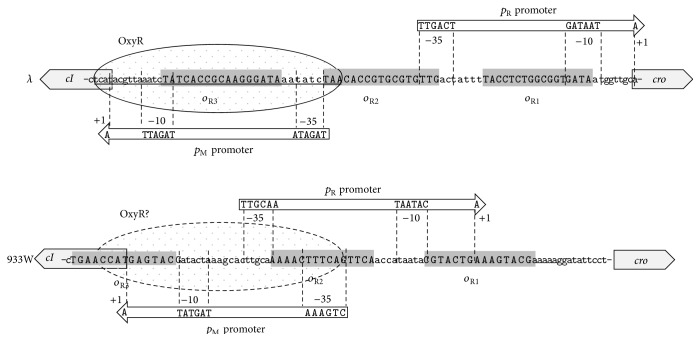
The *p*_R_-*p*_M_ regions of phage *λ* and Shiga toxin-converting phage 933W. Structural elements of each promoter are indicated, and *o*_R1_, *o*_R2_, and *o*_R3_ operator sequences are marked. The OxyR binding to the *λp*_R_-*p*_M_ region (demonstrated experimentally [26]) is shown as a solid oval, and a putative (not verified experimentally) OxyR binding to the 933W *p*_R_-*p*_M_ region is suggested by a dashed oval.

**Figure 4:**
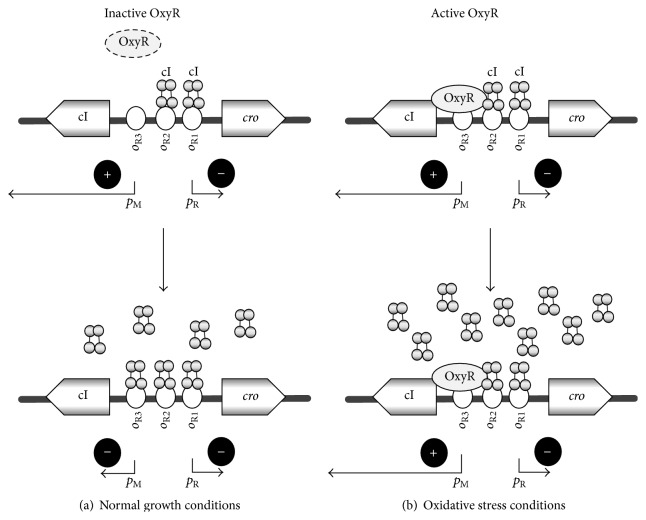
A model for OxyR-mediated modulation of lambdoid prophage maintenance under normal growth conditions and oxidative stress. Under normal growth conditions (a), the OxyR protein is inactive. The cI protein binds to *o*_R1_ and *o*_R2_ operators, repressing *p*_R_ and stimulating *p*_M_. At high concentrations, cI binds also to *o*_R3_ which caused repression of *p*_M_. Under oxidative stress conditions (b), OxyR is activated and binds to the *o*_R3_ region. This stimulates binding of cI to *o*_R1_ and *o*_R2_, enhancing *p*_R_ repression and *p*_M_ stimulation, and when cI concentration increases, *p*_M_ repression is prevented by competitive binding to *o*_R3_ by OxyR. This results in higher activity of *p*_M_ than that under normal growth conditions, increased levels of cI, and more efficient maintenance of the prophage.

It was demonstrated that the prophage induction by hydrogen peroxide is over 100 times more effective in cells with deletion of the* oxyR* gene than in wild-type control [26]. The OxyR protein is a transcription factor acting as a major regulator of the oxidative stress [34]. In the phage DNA region responsible for the control of maintenance of the prophage, there are 3 sites for binding of the cI repressor, called *o*_R1_, *o*_R2_, and *o*_R3_ (Figure 3). Binding of the cI protein to *o*_R1_ and *o*_R2_ represses *p*_R_, the major promoter for expression of genes required during the lytic development, but at the same time stimulates transcription of the* c*I gene from the *p*_M_ promoter. At high concentrations of cI, this protein can bind also to *o*_R3_ which causes a repression of its own promoter *p*_M_ [20] (Figure 4).

Detailed molecular studies indicated that OxyR can bind specifically to the region of the *p*_M_-*p*_R_ promoters (introduction of mutations to the putative OxyR-binding site abolished interactions of this protein with DNA), though with a weaker affinity than to its own promoter [26]. Interestingly, in the presence of OxyR, the cI protein interactions with *o*_R1_, *o*_R2_ were enhanced, while binding of this repressor to *o*_R3_ was impaired. These results suggested that OxyR might stimulate repression of *p*_R_ and activation of *p*_M_ but at the same time downregulate repression of *p*_M_ [26]. This would lead to a considerably more efficient maintenance of the prophage due to more efficient blocking of the *p*_R_ promoter by abundant cI. Indeed, studies with gene fusions showed that while under normal growth conditions (when OxyR is inactive) the activity of the *p*_M_ promoter was similar in both* oxyR* ^+^ and Δ*oxyR* strains, the oxidative stress conditions (treatment of cells with H_2_O_2_ which activates OxyR) caused enhanced transcription from *p*_M_ in wild-type bacteria and decreased in the Δ*oxyR* mutant [26]. Therefore, it appears that the OxyR protein is responsible for the low efficiency of prophage induction caused under conditions of the oxidative stress (Figure 4).

## 3. The Oxidative Stress and Biological Role of Shiga Toxin Production by STEC

There is an intriguing question regarding the biological role of Shiga toxin production by STEC strains. As described in preceding sections, expression of* stx* genes is effective only after Shiga toxin-converting prophage induction. However, this also results in subsequent lytic development of the bacteriophage and eventual death of the host cell. Thus, what can be a benefit for the bacterium from production of Shiga toxin while it is linked to its death? On the other hand, if toxin production was not beneficial for* E. coli*, one should expect a positive selection of lysogenic bacteria with mutations causing deficiency in prophage induction and thus elimination of STEC cells from the bacterial population. Since this is not the case, it should be beneficial for STEC to produce Shiga toxin.

It was suggested that STEC virulence in humans may be coincidental with the biological role for Shiga toxin being unrelated to human infection [35]. This hypothesis assumed that synthesis of Shiga toxins by STEC may enhance survival of bacteria in food vacuoles of protozoan predators. Interestingly, such a phenomenon was demonstrated experimentally [36]. Moreover, a bacterivorous, protozoan predator,* Tetrahymena thermophila*, was shown to be killed when cocultured with bacteria lysogenic with Stx bacteriophage [37]. However, this killing did not occur in the presence of catalase, an enzyme responsible for hydrogen peroxide breakdown [37]. In fact,* Tetrahymena* produces H_2_O_2_ to damage bacterial cells during attack by this predator [37]. This may be a successful predatory strategy in the case of the vast majority of bacteria; however, if STEC cells are being attacked, Shiga toxin-converting prophages are induced due to action of hydrogen peroxide (as demonstrated experimentally [30, 31]), Shiga toxin is produced, and after toxin release from* E. coli* due to phage-mediated cell lysis, it kills the predator. The crucial point of such a defensive bacterial strategy is a low effective prophage induction by H_2_O_2_ which has also been shown [30, 31]. Therefore, of the total STEC population, only 1% or less is lost for production of Shiga toxin (which is enough to produce relatively large amounts of the toxin, sufficient to kill the predator) while the rest of bacteria are saved. When STEC infects human intestine, neutrophils' action is similar to that of protist predators, and H_2_O_2_ is produced to kill bacteria [38], but the effects are analogous to the* Tetrahymena*-STEC interplay. The hypothesis on such an “bacterial altruism” has been proposed independently by two groups [17, 39], and detailed analyses of the literature indicated that the predicted scenario may be true [32]. Moreover, the hypothesis has been further confirmed by recent discoveries that STEC strains are more resistant to the impact of grazing protists than* E. coli* devoid of the* stx* genes [40] and that bacteriophage-mediated lysis of STEC is necessary for killing of protist cells by Shiga toxin, since the toxin released as a consequence of digestion of bacteria by* Tetrahymena* is harmless to it [41]. The latter finding was the argument to call the STEC cells a “Trojan Horse,” carrying genes encoding the toxin into target organisms [42].

## 4. Concluding Remarks

The oxidative stress plays a pivotal role in the production of Shiga toxins in cells of enterohemorrhagic* Escherichia coli* (EHEC) infecting human intestine, as well as in response to the attack of predator protists. In both cases, hydrogen peroxide is excreted by eukaryotic cells (either protist predators or neutrophils in an infected organism) to weaken bacteria which is a successful strategy against most prokaryotes; however, EHEC strains are lysogenic for Shiga toxin-converting prophages, and H_2_O_2_ stimulates their induction. This leads to the switch to lytic development and production of the toxin. It appears that Shiga toxin-producing bacteria use the specific strategy of “bacterial altruism,” based on the OxyR-mediated low efficiency of prophage induction during the oxidative stress. As a consequence, only a small fraction of bacterial cells is destroyed due to prophage induction, which is nevertheless sufficient to produce relatively large amounts of Shiga toxins able to kill eukaryotic cells. In this way the rest of the* E. coli* population can survive the attack of the predator or neutrophils.

## References

[1] Hartl D. L., Dykhuizen D. E. (1984). The population genetics of *Escherichia coli*. *Annual Review of Genetics*.

[2] Gyles C. L. (2007). Shiga toxin-producing *Escherichia coli*: an overview. *Journal of Animal Science*.

[3] Hunt J. M. (2010). Shiga toxin-producing *Escherichia coli* (STEC). *Clinics in Laboratory Medicine*.

[4] Razzaq S. (2006). Hemolytic uremic syndrome: an emerging health risk. *American Family Physician*.

[5] Law D. (2000). Virulence factors of *Escherichia coli* O157 and other Shiga toxin- producing *E. coli*. *Journal of Applied Microbiology*.

[6] LaPointe P., Wei X., Gariépy J. (2005). A role for the protease-sensitive loop region of Shiga-like toxin 1 in the retrotranslocation of its A1 domain from the endoplasmic reticulum lumen. *The Journal of Biological Chemistry*.

[7] Tam P. J., Lingwood C. A. (2007). Membrane-cytosolic translocation of verotoxin A_1_ subunit in target cells. *Microbiology*.

[8] Obrig T. G., Moran T. P., Brown J. E. (1987). The mode of action of Shiga toxin on peptide elongation of eukaryotic protein synthesis. *Biochemical Journal*.

[9] Endo Y., Tsurugi K., Yutsudo T., Takeda Y., Ogasawara T., Igarashi K. (1988). Site of action of a vero toxin (VT2) from *Escherichia coli* O157:H7 and of Shiga toxin on eukaryotic ribosomes. RNA N-glycosidase activity of the toxins. *European Journal of Biochemistry*.

[10] Beutin L., Martin A. (2012). Outbreak of shiga toxin-producing *Escherichia coli* (STEC) O104:H4 infection in Germany causes a paradigm shift with regard to human pathogenicity of STEC strains. *Journal of Food Protection*.

[11] Bloch S. K., Felczykowska A., Nejman-Falenczyk B. (2012). *Escherichia coli* O104:H4 outbreak—have we learnt a lesson from it?. *Acta Biochimica Polonica*.

[12] Karch H., Denamur E., Dobrindt U. (2012). The enemy within us: lessons from the 2011 European *Escherichia coli* O104:H4 outbreak. *EMBO Molecular Medicine*.

[13] Buchholz U., Bernard H., Werber D. (2011). German outbreak of *Escherichia coli* O104:H4 associated with sprouts. *The New England Journal of Medicine*.

[14] Mellmann A., Harmsen D., Cummings C. A. (2011). Prospective genomic characterization of the German enterohemorrhagic *Escherichia coli* O104:H4 outbreak by rapid next generation sequencing technology. *PLoS ONE*.

[15] Werber D., Krause G., Frank C. (2012). Outbreaks of virulent diarrheagenic *Escherichia coli*—are we in control?. *BMC Medicine*.

[16] Allison H. E. (2007). Stx-phages: drivers and mediators of the evolution of STEC and STEC-like pathogens. *Future Microbiology*.

[17] Łoś J. M., Łoś M., Wegrzyn G. (2011). Bacteriophages carrying Shiga toxin genes: genomic variations, detection and potential treatment of pathogenic bacteria. *Future Microbiology*.

[18] Johansen B. K., Wasteson Y., Granum P. E., Brynestad S. (2001). Mosaic structure of Shiga-toxin-2-encoding phages isolated from *Escherichia coli* O157:H7 indicates frequent gene exchange between lambdoid phage genomes. *Microbiology*.

[19] Ptashne M. (2004). *A Genetic Switch, Phage Lambda Revisited*.

[20] Węgrzyn G., Węgrzyn A. (2005). Genetic switches during bacteriophage *λ* development. *Progress in Nucleic Acid Research and Molecular Biology*.

[21] Wegrzyn G., Licznerska K., Wegrzyn A. (2012). Phage *λ*-new insights into regulatory circuits. *Advances in Virus Research*.

[22] Imamovic L., Muniesa M. (2012). Characterizing RecA-independent induction of Shiga toxin2-encoding phages by EDTA treatment. *PLoS ONE*.

[23] Koudelka A. P., Hufnagel L. A., Koudelka G. B. (2004). Purification and characterization of the repressor of the shiga toxin-encoding bacteriophage 933W: DNA binding, gene regulation, and autocleavage. *Journal of Bacteriology*.

[24] Serra-Moreno R., Jofre J., Muniesa M. (2008). The CI repressors of Shiga toxin-converting prophages are involved in coinfection of Escherichia coli strains, which causes a down regulation in the production of Shiga toxin 2. *Journal of Bacteriology*.

[25] Ren J., Wen L., Gao X., Jin C., Xue Y., Yao X. (2009). DOG 1.0: illustrator of protein domain structures. *Cell Research*.

[26] Glinkowska M., Łoś J. M., Szambowska A. (2010). Influence of the *Escherichia coli* oxyR gene function on lambda prophage maintenance. *Archives of Microbiology*.

[27] Yamamoto T., Kojio S., Taneike I., Nakagawa S., Iwakura N., Wakisaka-Saito N. (2003). ^60^Co irradiation of Shiga toxin (Stx)-producing *Escherichia coli* induces Stx phage. *FEMS Microbiology Letters*.

[28] Aertsen A., Faster D., Michiels C. W. (2005). Induction of Shiga toxin-converting prophage in *Escherichia coli* by high hydrostatic pressure. *Applied and Environmental Microbiology*.

[29] Wagner P. L., Acheson D. W. K., Waldor M. K. (2001). Human neutrophils and their products induce Shiga toxin production by enterohemorrhagic *Escherichia coli*. *Infection and Immunity*.

[30] Łoś J. M., Łoś M., Węgrzyn G., Węgrzyn A. (2009). Differential efficiency of induction of various lambdoid prophages responsible for production of Shiga toxins in response to different induction agents. *Microbial Pathogenesis*.

[31] Łoś J. M., Łoś M., Wȩgrzyn A., Wȩgrzyn G. (2010). Hydrogen peroxide-mediated induction of the Shiga toxin-converting lambdoid prophage ST2-8624 in *Escherichia coli* O157:H7. *FEMS Immunology and Medical Microbiology*.

[32] Loś J. M., Loś M., Wegrzyn A., Wegrzyn G. (2013). Altruism of Shiga toxin-producing *Escherichia coli*: recent hypothesis versus experimental results. *Frontiers in Cellular and Infection Microbiology*.

[33] Nejman B., Łoś J. M., Łoś M., Wgrzyn G., Wgrzyn A. (2009). Plasmids derived from lambdoid bacteriophages as models for studying replication of mobile genetic elements responsible for the production of shiga toxins by pathogenic *Escherichia coli* strains. *Journal of Molecular Microbiology and Biotechnology*.

[34] Chiang S. M., Schellhorn H. E. (2012). Regulators of oxidative stress response genes in *Escherichia coli* and their functional conservation in bacteria. *Archives of Biochemistry and Biophysics*.

[35] Brandl M. T. (2006). Fitness of human enteric pathogens on plants and implications for food safety. *Annual Review of Phytopathology*.

[36] Steinberg K. M., Levin B. R. (2007). Grazing protozoa and the evolution of the *Escherichia coli* O157:H7 Shiga toxin-encoding prophage. *Proceedings of the Royal Society B: Biological Sciences*.

[37] Lainhart W., Stolfa G., Koudelka G. B. (2009). Shiga toxin as a bacterial defense against a eukaryotic predator, *Tetrahymena thermophila*. *Journal of Bacteriology*.

[38] Tsan M. F., Douglass K. H., McIntyre P. A. (1977). Hydrogen peroxide production and killing of *Staphylococcus aureus* by human polymorphonuclear leukocytes. *Blood*.

[39] Mauro S. A., Koudelka G. B. (2011). Shiga toxin: expression, distribution, and its role in the environment. *Toxins*.

[40] Mauro S. A., Opalko H., Lindsay K., Colon M. P., Koudelka G. B. (2013). The microcosm mediates the persistence of shiga toxin-producing *Escherichia coli* in freshwater ecosystems. *Applied and Environmental Microbiology*.

[41] Stolfa G., Koudelka G. B. (2013). Entry and killing of *Tetrahymena thermophila* by bacterially produced Shiga toxin. *mBio*.

[42] Arnold J. W., Koudelka G. B. (2014). The Trojan Horse of the microbiological arms race: phage-encoded toxins as a defence against eukaryotic predators. *Environmental Microbiology*.

